# The presence of lupus nephritis additionally increases the risk of preeclampsia among pregnant women with systemic lupus erythematosus

**DOI:** 10.1177/09612033211004716

**Published:** 2021-04-12

**Authors:** Katarina Bremme, Sonja Honkanen, Iva Gunnarsson, Roza Chaireti

**Affiliations:** 1 Department of Women's and Children's Health, Karolinska Institutet, Stockholm, Sweden; 2 Division of Obstetrics and Gynaecology, Karolinska University Hospital, Stockholm, Sweden; 3 Division of Rheumatology, Department of Medicine, Karolinska Institutet, Stockholm, Sweden; 4 Division of Rheumatology, Karolinska University Hospital, Stockholm, Sweden; 5 Department of Molecular Medicine and Surgery, Karolinska Institute, Solna, Sweden; 6 Department of Hematology, Karolinska University Hospital, Stockholm, Sweden

**Keywords:** Systemic lupus erythematosus, lupus nephritis, obstetric outcome, preeclampsia, premature birth

## Abstract

**Introduction:**

Pregnant women with systematic lupus erythematosus (SLE) have an increased risk of obstetric complications, such as preeclampsia and premature births. Previous studies have suggested that renal involvement could further increase the risk for adverse obstetric outcomes. *Aims:* The aim of this study was to compare the obstetric outcomes in a Swedish cohort of patients with SLE with and without lupus nephritis (LN).

**Patients and methods:**

The study was conducted as a retrospective observational study on 103 women with SLE, who gave birth at the Karolinska University Hospital between the years 2000-2017. Thirty-five women had previous or active LN and 68 women had non-renal lupus. Data was collected from digital medical records. The outcomes that were analysed included infants born small for gestational age (SGA), premature birth, preeclampsia, SLE- or nephritis flare and caesarean section.

**Results:**

Women with LN, both with previous and with renal flare during pregnancy suffered from pre-eclampsia more often compared to women with non-renal lupus (25.7% vs 2.9%, p = 0.001) and this complication was associated with premature birth (p = 0.021) and caesarean section (p = 0.035).

**Conclusions:**

Lupus nephritis is a significant risk factor for adverse obstetric outcomes in women with SLE, including preeclampsia. Those patients could benefit from more frequent antenatal controls and more vigorous follow-up.

## Introduction

Pregnancies in women with systemic lupus erythematosus (SLE) are considered as entailing a very high risk for complications, such as preeclampsia, miscarriages, stillbirths and preterm births, as well as thromboembolism.^1,2^ Women with SLE are additionally delivered by caesarean section (c-section) more frequently compared to pregnant women without SLE.^2^ Children born to mothers with SLE have a higher risk of being small for gestational age (SGA) and having lower APGAR scores.^1,2^ Furthermore, maternal autoantibodies can be transferred to the foetus and cause neonatal lupus.^3^

Lupus nephritis (LN) is a relatively common and severe manifestation of SLE, with increased mortality and morbidity.^4^ There is some but limited data suggesting that LN increases the risk for obstetric complications.^5^ A study by Moroni et al. on 71 pregnancies showed an increase in maternal complications [renal flare, preeclampsia, Hemolysis Elevated Liver enzymes Low Platelet syndrome (HELLP)] in women with LN, but concluded that even other, unrelated factors such as immunological activity, high blood pressure and high body mass index (BMI) may contribute to this increased risk.^6^ Another study on the same cohort showed that a high rate of preterm deliveries associated with LN flares, pre-eclampsia and high disease activity during pregnancy, as well as a high frequency of children born SGA.^7^

Although previous data from different populations suggest that pregnancies in women with LN have an additionally increased risk to develop obstetric complications,^8,9^ no Swedish studies have been conducted. Considering the geographical and ethnic variations in the prevalence, presentation as well as treatment of SLE,^10,11^ it is of interest to confirm previous findings and to investigate the association between LN and obstetric (maternal and foetal) outcomes in a Swedish (mainly Caucasian) population.

## Patients and methods

### Patient cohort and inclusion process

A single-centre, retrospective, observational study was conducted by reviewing the digital medical records of 103 pregnant women with SLE during the years 2000-2017. Inclusion criteria were: i) confirmed SLE diagnosis (according to the criteria described by Tan et al.^12^) ii) live birth and iii) both pregnancy- and SLE-related check-ups, as well as delivery, conducted at the Karolinska University Hospital in Stockholm, Sweden. The study was approved by the Regional Ethics Committee (Stockholm). All patients had given their written consent prior to inclusion in the study.

The diagnosis of LN is set based on clinical symptoms according to the criteria issued by the American College of Rheumatology (ACR)^12^ and the results of a renal biopsy in most cases. For patients with a renal biopsy, the different classes were classified according to the criteria established by the International Society of Nephrology/Renal Pathology Society (ISN/RPS).^13^ The patients who met the inclusion criteria were divided into two groups based on the occurrence or absence of LN. If a woman had had several pregnancies during the study period, only the first pregnancy was included (one pregnancy/patient). We chose not include subsequent pregnancies in order to better study the patient management prior to medical adjustments due to previous adverse obstetric events. Of the 112 patients fulfilling the criteria, 7 were excluded because they were lost to follow-up or gave birth at a different hospital and two because they had twin pregnancies, which are high risk pregnancies. In total, 103 patients (103 pregnancies) were included in the study.

### Data collected from medical records and laboratory results

The study data included: i) demographics (age, BMI), ii) data on the current and previous pregnancies (delivery week, number of pregnancies and deliveries, miscarriages), iii) maternal and foetal outcomes (SGA, premature birth, preeclampsia, mode of delivery including c-section, APGAR score) and iv) data on SLE disease and LN (flares, duration of disease, medications, proteinuria).

The Systemic Lupus Erythematosus Disease Activity Index 2000 (SLEDAI-2K) was calculated as previously described.^14^ We used a cut-off of >4 to define active disease, in accordance to previous studies.^15^ Organ damage was calculated according to the Systemic Lupus International Collaborating Clinics Damage index (SLICC-DI).^16^ SELDAI-2K was used to characterize disease activity within 6 months prior to and at conception, SLICC-DI was estimated at onset of pregnancy.

Data on obstetric and neonatal outcomes extracted from the National (Swedish) statistical database on pregnancies, deliveries and newborns published by the National Board of Health and Welfare were used as controls.^17^ We thus used data from pregnancies, deliveries and newborns for all women giving birth in Stockholm County between the years 2000 and 2016, i.e. the timeframe of the study. Data from 2017 were not available at the time of the study and the subsequent statistical analysis.

### Definitions

Preeclampsia was defined as gestational hypertension and newly onset of proteinuria, ≥300 mg/24 hours after 20 weeks of gestation.^18^

Gestational hypertension was defined as blood pressure ≥ 140/90 mmHg.^18^

The newborns were considered SGA if they had a weight and/or length of at least 2 standard deviations (SD) below the mean for gestational age.^19^

A SLE flare was defined as a clinically significant increase in disease activity, requiring change or escalation of therapy.^20^

A LN flare was defined as a significant increase in proteinuria or serum creatinine, or a reduction in creatinine clearance, and/or an abnormal urinary sediment due to active disease.^20^

### Laboratory variables

Creatinine was measured by a photometric method and expressed as µmol/L (reference interval <90 µmol/L for women). The estimated glomerular filtration rate (eGFR) was calculated according to the Lund-Malmö revised formula, which is based on the creatinine value and takes the body mass into consideration, and is expressed in mL/min/1,73 m^2^ (reference >90 mL/min/1,73 m^2^).^21^ No separate reference interval for creatinine and eGFR for pregnant women was available at the Karolinska University Hospital laboratory.

### Statistics

Data was analysed using the IBM SPSS Statistics 24 program for Windows. Continuous variables were tested for normality using the Shapiro-Wilk test. Continuous data was presented as mean and standard deviation or as median and range (min-max) depending on the distribution. For normally distributed data the student´s t-test for independent sample was used and for not normally distributed data the Mann-Whitney U-test was applied to compare the two groups LN and non-LN. Categorical data were summarized using frequency counts and percentages and analysed with Fisher’s exact test (using 2-sided significance testing). In addition, logistic regression model was used to analyse association of different maternal factors on obstetric outcomes. A p-value < 0.05 was considered significant.

## Results

### Study population

Of the 103 patients included, 35 had either a history of LN or active LN. Lupus nephritis diagnosis was confirmed by a renal biopsy in a majority of cases (31/35, 88.6%). The remaining 68 patients were included in the group with non-renal SLE. Three of the women in the cohort were first diagnosed with SLE during pregnancy, of whom one had LN.

SLEDAI and SLICC-DI scores were not calculated for the three patients who were diagnosed with SLE during pregnancy, and additionally for nine patients due to missing data. Of the 91 patients where the scores were calculated, two (both with LN) had SLEDAI > 4 during the six months period prior to conception. Twelve (two with LN and ten without LN) had a SLEDAI score of 2 and thirteen (six with LN and seven without LN) had an index of 3-4, thus most patients were in an inactive phase of disease.

The presence of flares prior to conception could similarly not be evaluated in nine patients and was not relevant in three, as mentioned above. Three patients with LN had flares (mainly arthritis) prior to conception. No patients had diabetes mellitus and seven patients with LN had been previously diagnosed with chronic arterial hypertension and were using antihypertensive drugs prior to conception. Seven patients with a previous history of nephritis had low-grade proteinuria (<0.3 g/day) 6 months before conception and were all regarded to be in renal remission.

No significant difference was found between the two groups regarding the baseline characteristics (Table 1). The mean ± SD age of all mothers at delivery was 31.9 ± 4.4 years. The majority (84.5%) of the women were nulligravida. Twenty-seven out of 103 (26.5%) women had had a previous miscarriage. Arterial hypertension was found in two patients (1.9%) at the start of pregnancy, both with LN. Proteinuria was present at the start of pregnancy in fourteen women in the LN group (14/35 patients, 40%). At the onset of pregnancy, patients with LN had higher creatinine values [58 (38-148) mmol/L and 50 (37-87) mmol/L respectively, p = 0.023] and lower estimated glomerular filtration rates (eGFR) [9111.5 (72–129) ml/min/1,73 m^2^and 98.8 (34–129) ml/min/1,73 m^2^, respectively, p = 0.008] (Table 1) compared to the women without LN.

**Table 1.** Demographic, disease and early pregnancy characteristics of the patients included in the study.

Characteristics	All patients	Non-LN (*n* = 68)	LN (*n* = 35)	*p*
Duration of SLE in years	9 (0–28)	8.5 (0–23)	9 (0–28)	0.398
Age of first-time mothers	31.6 ± 4.5	32.0 ± 5.0	30.8 ± 3.1	0.193
BMI^a^	23.0 (18–44)	22.5 (19–44)	23.0 (18–31)	0.639
Nulliparity	87/103 (84.5%)	59/68 (86.8%)	28/35 (80.0%)	0.398
Previous miscarriage	27/102 (26.5%)	17/67 (25.4%)	10/35 (28.6%)	0.814
Treatment	All patients	Non-LN (*n* = 68)	LN (*n* = 35)	*p*
Aspirin	61 (59.2%)	33 (48.5%)	28 (80.0%)	0.003*
Prednisolone	49 (26.2%)	26 (38.2%)	23 (65.7%)	0.012*
Hydroxychloroquine	40 (38.9%)	25 (36.8%)	15 (42.9%)	0.670
Azathioprine	17 (16.5%)	7 (10.3%)	10 (28.6%)	0.025*
LMWH	19 (18.4%)	9 (13.2%)	10 (28.6%)	0.066
No treatment	17 (16.5%)	15 (22.1%)	2 (5.7%)	0.048*
Early pregnancy^b^	All patients non-LN (*n* = 68) LN (*n* = 35)			*p*
Systolic BP (mm Hg)	110 (92–140)	110 (92–130)	110 (95–140)	0.497
Diastolic BP (mm Hg)	70 (50–95)	70 (50–85)	65 (50–95)	0.693
P-creatinine (mmol/l)	52 (37–148)	50 (37–87)	58 (38–148)	0.023*
Proteinuria	14 (13.6%)	0 (0.0%)	14 (40.0%)	0.000*
eGFR (ml/min/1.73 m^2^)	108.7 (34-129)	111.5 (72–129)	98.8 (34–129)	0.008*

LMWH: low molecular weight heparin.

Age presented as mean ± SD, comparison with independent *t* test; body mass index (BMI), disease duration presented as median (min-max), comparison with Mann–Whitney *U* test; categorical variables analysed with Fisher’s exact test.

Blood pressure (BP), creatinine and eGFR (calculated by the Cockroft-Gault formula) presented as median (min-max), comparison with Mann–Whitney *U* test; categorical variables analysed with Fisher’s exact test.

Statistically significant results marked with *. *p* values refer to comparisons between non-LN and LN groups.

^a^At first visit to maternity clinic (*n* = 81).

^b^Data from the first control at the maternity clinic.

### Characteristics of treatment

**Figure 1.**
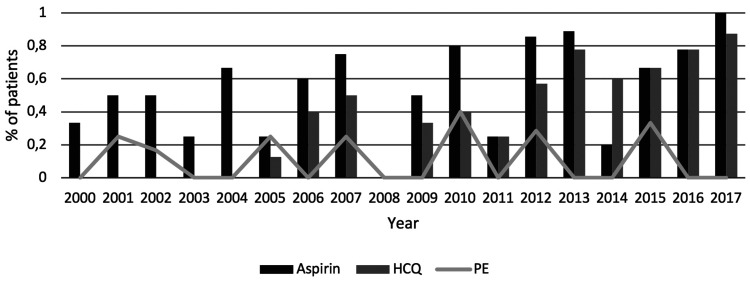
Differences in treatment with aspirin and hydroxychloroquine (HCQ) over study period presented with concurrent preeclampsia (PE) cases.

Antenatal treatment with aspirin (75 mg), corticosteroids and azathioprine was more common in the LN group (22%) compared to the non-renal group (6%) (p = 0.003, p = 0.012 and p = 0.025, respectively) (Table 1). Out of the 49 patients treated with corticosteroids antenatally, data on dosage were available for 40 17 with and 23 without LN, median dosage (5mg (2–10 mg) and 5 mg (2.5–17.5) respectively). The usage of aspirin and hydroxychloroquine (HCQ) increased significantly during the study period. According to updated treatment recommendations, HCQ was first administered to a patient in our cohort in 2005. Combination therapy with aspirin and HCQ became likewise more common towards the end of the study period concurrently with reduction in the rates of preeclampsia (Figure 1).

### Histopathological findings in the LN cohort

Results from kidney biopsies were available in 31/35 patients with LN. Some of the patients had undergone multiple biopsies, and for the characterization of the histopathological findings in cases with more than one biopsy, we used the most severe ISN/RPS class. Among the patients who had a renal biopsy, 5 had class II (16%), 7 class III (23%), 15 class IV (48%) and 4 class V (13%).

One of the women included in the LN group had previously developed end-stage renal failure and had received a kidney transplant.

### Obstetric outcomes and complications

The majority (33/35, 94%) of the patients had developed LN prior to the pregnancy and the remaining two presented with LN during the pregnancy. One of the women without a previous SLE diagnosis presented with nephrotic syndrome late in the second trimester. The diagnosis (SLE with LN) was confirmed by renal biopsy a few months after delivery. The other case of LN initially diagnosed during pregnancy was observed in a patient with confirmed SLE, with a biopsy performed a few years after delivery.

Twenty-six patients (26/103, 25%) had a SLE flare during pregnancy (16 in the non-LN group and 10 in the LN group, p = 0.635). Six patients (6/103, 6%) developed renal flares, two of which occurred in patients without previous LN, as described above.

Women in the LN group experienced preeclampsia at a higher rate, 25.7% (9/35 patients) compared to 2.9% (2/68 patients, p = 0.001) in the non-LN group. Premature births (<37 weeks) were more common in the LN group, seen in 9 of 35 (25.7%) in the LN group compared to 5 of 67 (7.5%, p = 0.016) in the non-LN group. SGA and c-sections did not show any significant difference between the two groups (Table 2). Preeclampsia was observed in 6 of 9 (66.7%) preterm deliveries in women with LN. Two children were born with neonatal lupus.

**Table 2.** Obstetric outcomes in study population, non-renal lupus patients and lupus nephritis patients.

	Stockholm County^a^	All (*N*)	Non-LN	LN	
Outcomes	Mean±SD	M Median (min-max)	Median (min-max)	Median (min-max)	*p*
Gestational age (weeks)	N/A	39 (25–42)	39 (34–42)	38 (25–42)	0.150
Birth weight (g)	3489 ± 19.7	3115 (498–4615)**	3225 (2150–4615)	3030 (498–4160)	0.252
	Mean %±SD%	*n*/*N* (%)	*n*/*N* (%)	*n*/*N* (%)	*p*
Preeclampsia	N/A	11/103 (10.7%)	2/68 (2.9%)	9/35 (25.7%)	0.001*
Premature birth (before w.37)	2.9%± 0.1%	14/102 (13.7%)	5/67 (7.5%)	9/35 (25.6%)	0.016*
Caesarean section	20.0% ± 1.4%	34/103 (33.0%)	23/68 (33.8%)	11/35 (31.4%)	1.000
SGA	2.4% ± 0.14%	12/103 (11.6%)	7/68 (10.3%)	5/35 (14.3%)	0.536
APGAR < 7 (1 min)	N/A	9/97 (9.3%)	7/63 (11%)	2/34 (5.9%)	0.487
APGAR < 7 (5 min)	1.0% ± 0.1%	1/97 (1.0%)	1/65 (1.5%)	0/32 (0%)	1.000

N/A: not available; SGA: small for gestational age.

Mann–Whitney *U* test used for continuous variables and Fisher’s exact test for categorical variables.

*Statistically significant. *p* values refer to comparisons between non-LN and LN groups.

**Mean: 3092 ± 631 (for comparison to controls).

^a^Data from all pregnancies and deliveries in Stockholm County, 2000–2016.^17^

According to the results of a crude logistic regression analysis, kidney function variables (proteinuria, eGFR < 90 ml/min/1,73 m^2^), and the use of different drugs [aspirin, low molecular weight heparin (LMWH), HCQ, azathioprine and corticosteroids] were not significantly associated with children born SGA and c-sections. Proteinuria was the only variable associated with preeclampsia and premature births (data not shown).

Using the logistic regression method, we analysed the effect of maternal age, SLE disease duration at the beginning of pregnancy, previous nephritis, renal flares, other SLE flares, nulliparity, chronic arterial hypertension, previous thrombosis, active urine sediment within 6 months prior to conception, as well as use of aspirin, HCQ and LMWH on preeclampsia. It was shown that preeclampsia was associated with renal flares (p = 0.004) and longer duration of SLE (p = 0.019). When adjusted for BMI, the association of preeclampsia with disease duration was no longer significant (p = 0.326), whereas the association with renal flares remained significant (p = 0.029).

A similar analysis was performed on the effect of age, disease duration, preeclampsia, renal flares, other lupus flares, pre-existing nephritis, chronic arterial hypertension, previous thrombosis, active urine sediment within 6 months prior to conception and immunosuppressive treatment on premature birth and c-sections. Preeclampsia was the only factor significantly associated with both outcomes (p = 0.001 for premature births and p = 0.009 for c-sections).

## Discussion

This single-centre study on a SLE cohort indicates a significantly increased risk for preeclampsia in women with SLE and renal involvement. Preeclampsia was subsequently associated with renal flares, c-sections and premature birth. The presence of SLE with LN was thus shown to increase the risk for obstetric complications.

The incidence of preeclampsia in the general population is approximately 3%.^22^ In the study cohort, women without LN developed preeclampsia at a rate of 2.9%, in a rate observed in healthy women. Of note, women with LN developed preeclampsia almost nine times as often (25.6%). Furthermore, in the LN group, 8 of 9 (89%) women with preeclampsia belonged to ISN/RPS class III/IV, in line with data from a recent article by Rodrigues et al.,^23^ which showed a similar association between more advanced LN grade and obstetric complications.

In a prospective study,^6^ pregnant patients with LN developed preeclampsia at a rate of 8.4%, which is lower compared to the rate reported in our cohort (25.7%). The patient groups are not, however, entirely comparable, as we included both women with pre-existing and new-onset LN in the nephritis group. Furthermore, there were some differences in the pharmacological treatment between our study and the study by Moroni et al.^6^ They also included more than one pregnancy in the same woman. Interestingly, both studies showed an association between SLE duration and preeclampsia. Another study reported higher rates of preeclampsia in women both with and without previous LN (28% vs 16%).^24^

In line with previous studies,^2,24^ we observed that more pregnancies in women with SLE were delivered by c-section compared to the average rate of c-sections in Stockholm County during the same time period (33% vs. 20%). In one of the aforementioned studies (a meta-analysis), pregnant women with SLE delivered by c-section almost twice as often as the control group.^2^ In the other study, 65% of all women both with and without previous lupus nephritis, were delivered by c-section.^24^ As expected, preeclampsia was associated with c-sections in the present study.

The newborns in our cohort were generally in good health, with similar rates of APGAR scores compared to all the infants born in Stockholm County.^17^ However, infants born to SLE patients were premature at a much higher frequency compared to the average rates of premature births in Stockholm.^17^ This is of particular importance as prematurity can cause both short and long-term complications.^8^ Furthermore, patients with LN had over a three-fold risk for delivering prematurely compared to non-LN mothers. These results are in accordance with the rates of premature birth (28.2%) reported in a previous prospective study,^7^ where preterm birth was associated to nephritis flares, preeclampsia and high disease activity during pregnancy. In the present study, preeclampsia was likewise associated to prematurity and nephritis flares. In a previous meta-analysis both LN and SLE activity preconceptionally were recognised as risk factors for premature birth, especially in mothers with active LN.^8^

The newborn infants of SLE mothers were approximately 500 grams lighter at birth compared to the babies born in Stockholm County between 2000-2016.^17^ The risk of SGA was generally higher in patients with SLE^2^ with an observed rate of 11.6% in the study population compared to the average of 2.4% of babies born SGA in Stockholm.^17^ A prospective study on pregnant women with LN showed similar rates of children born SGA (16.4%).^7^ No statistically significant differences were found when comparing infant birthweight and rates of children born SGA between mothers with different LN classes (data not shown). However, the median birth weight in women with LN class III/IV was lower compared to the other classes (2850 g and 3210 g respectively), in accordance to data presented in previous studies.^23^

All LN patients who developed preeclampsia had been on treatment with aspirin, which has previously been proved to reduce the risk for preeclampsia.^25^ A higher dosage has been suggested in certain high-risk groups^26,27^ and even if most centers still use a low dose (75-100 mg), higher dosages may be indicated in particular high-risk groups. During the course of the study, HCQ was gradually established for use during pregnancy,^28^ due to its positive effects on disease activity.^29^ We observed a reduction in preeclampsia rates and possibly fewer preterm births following broader usage of HCQ, which supports the recommendation to continue and/or initiate treatment with HCQ in all SLE pregnancies.^30^ The relation between active disease and obstetric complications is demonstrated by the association between renal flares and preeclampsia in this study, which points to the importance of adequate antenatal SLE control and patient compliance to treatment.

The size of our cohort is relatively large, considering the rarity of this condition. Since the study was conducted at a single hospital, all patients giving birth during the same time period would have had access to the same follow-up and have been treated in a similar manner, allowing for reliable comparisons among the groups. We additionally chose to compare neonatal outcomes such as SGA, APGAR and premature births in the study population with the outcomes in the larger Stockholm population during the same time period since the incidence of adverse outcomes varies internationally depending on, among others, socioeconomic factors. By choosing controls from the same population as the patients we ensure comparability of results. The vast majority of our patients did not have significant disease activity or flares prior to conception, which allowed us to study the outcomes without this confounding factor. However, the retrospective nature of the study sets some limitations on the availability of data and analysis. Due to the long study period, some of the methods used in diagnostics and treatments have changed, which could affect the comparability of the results during different time periods. Additionally, there were missing data on variables that might affect the rates of adverse obstetric outcomes, such as complement activation, smoking and family history of pre-eclampsia. Another limitation is the possible difficulty in defining a renal flare, as complications such as pre-eclampsia can mimic characteristics of this condition.

In conclusion, despite broader usage of medications such as HCQ and decrease in the rates of complications such as preeclampsia, pregnancies in women with SLE are still at high risk for obstetric complications. This was particularly obvious in patients with previous or active LN, where the risk for preeclampsia was significantly higher compared to both SLE patients without renal involvement and to the general population.
